# Relationship of Vertical Jump Performance and Ankle Joint Range of Motion: Effect of Knee Joint Angle and Handedness in Young Adult Handball Players

**DOI:** 10.3390/sports10060086

**Published:** 2022-05-28

**Authors:** Vassilios Panoutsakopoulos, Mariana C. Kotzamanidou, Athanasios K. Giannakos, Iraklis A. Kollias

**Affiliations:** 1 Biomechanics Laboratory, Department of Physical Education and Sports Science at Thessaloniki, Aristotle University of Thessaloniki, 54124 Thessaloniki, Greece; ayiannak@phed.auth.gr (A.K.G.); hkollias@phed.auth.gr (I.A.K.); 2 Faculty of Health Sciences, Metropolitan College of Thessaloniki, 54624 Thessaloniki, Greece; mkotzamanidou@metropolitan.edu.gr

**Keywords:** biomechanics, force parameters, video analysis, flexibility, throwing, laterality, symmetry, inter-segmental energy flow

## Abstract

The purpose of the study is to examine the effect of the ankle joint range of motion (ROM) on the vertical jump (VJ) performance of adult handball players. The active (ACT) and passive (PAS) ankle joint ROM of 12 male members of the U21 National Handball Team with the knee joint at 0°, 40°, and 90° flexion (0° = fully extended knee) was evaluated using a video analysis measuring method. Participants also performed maximum VJ with (CMJ) and without (SQJ) countermovement, as well as with (AS) and without (NAS) an arm swing. Statistical analyses included 2 × 2 × 3 MANOVA, 2 × 2 repeated measures ANOVA, and Pearson’s correlation. Results reveal that PAS-ROM was larger (*p* < 0.05) in all knee joint flexion angles. ROM was smaller (*p* < 0.05) by approximately 10° at 0° compared to 90° knee flexion. No lateral effects on ROM due to the handedness of the players were observed. AS and CM resulted in increased jump height (*p* < 0.05). Finally, ACT-ROM when the knee joint was flexed at 40° was highly correlated (*r* ≥ 0.66, *p* < 0.05) with VJ performance except for CMJ-AS. In conclusion, the differences in the bi-articular gastrocnemius muscle flexibility due to the alteration of the angular position of the examined joints affected the ability to generate impulse during the VJ tests.

## 1. Introduction

When executing a sports technique, the musculoskeletal system is obliged to confront the demands of the movement by applying the required forces to optimize performance. In indoor team sports, movement is conducted when the produced work from the muscles is transferred in a proximal to distal manner to the ground through the ankle joint [1]. The force and power production capabilities of athletes and their connection with sports performance are assessed using vertical jump tests [2]. For example, the concentric strength of the leg extensor muscles is evaluated using the squat jump (SQJ) [3]. Vertical jumps with a countermovement are used for testing the effectiveness of the utilization of the stretch–shortening cycle (SSC) [4], while the use of the arm swing leads to augmented mechanical work that can produce greater jump height given that the inter-segmental coordination facilitates the energy flow [5,6].

The ankle joint’s contribution to sports performance is dependent on the force that the surrounding acting muscles can apply and on its range of motion (ROM) [7]. Due to this, a large ankle joint ROM is important for the optimum execution of sports techniques and, subsequently, sports performance [8]. However, the large contribution of the ankle joint in the performance of indoor team sports, such as handball, leads to an increased occurrence of injury. This is evident by the fact that injury of the ligaments of the lower extremities [9,10,11] and ankle sprains in particular [12] are the most common injuries observed in handball players participating in major international competitions. In general, the majority of the related literature reports that the ankle is the joint mostly subjected to injury in handball players [13,14,15,16,17,18,19,20,21,22,23]. The outcome of the injuries occurring in the ankle joint is a reduced ROM and instability that cause decrements in handball players’ performance [14].

An inter-limb joint ROM difference of 6–8% was proposed to cause declined performance and increased injury risk in soccer players [24,25]. However, no inter-limb ankle ROM differences were found in untrained adults [26,27,28], Physical Education students [29], young female volleyball players [8], and professional soccer players [30,31]. In addition, ankle ROM was reported not to be different between lower extremities in young handball players [32,33]. Nevertheless, it is suggested that maturation has a significant effect on the flexibility of handball players as it is improved through adolescence [34,35,36].

The jump shot is the dominant throwing technique to score a goal in handball [37,38,39]. In general, the ankle joint plays a major role in jumping activities. The ankle joint plantar flexion at the push-off contributes to achieving about 22–23% of the take-off velocity [40,41]. This ankle joint contribution is defined by the force applied by the plantar flexors in the perspective of the temporal coincidence of their stimulation onset [42] and its ROM [43]. However, it is reported that when handball players perform a jump from the dominant leg, the jumping distance was approximately 5% larger than executing the jump with the non-dominant leg [44]. This can be attributed to the fact that the execution of the jump shot subjects the contralateral to the throwing arm leg to considerable mechanical loading [45,46,47,48]. Furthermore, long-term submission to systematic training results in sport-specific adaptations in strength and conditioning features [49], as well as to the factors that define handball performance [44].

Based on the above, it is of interest to examine if the preferred systematic use of an upper extremity to conduct throws such as the jump shot in handball results in inter-limb differences concerning the ankle ROM. To the best of the authors’ knowledge, there is a lack in the literature concerning the examination of asymmetry in lower limb parameters based on upper limb preference. The purpose of the study is to examine the active and passive ankle joint ROM in various knee joint flexion angles in young adult handball players. A secondary aim is to study the relationship of ankle joint ROM with performance in a variety of standardized vertical jump tests. It was hypothesized that the contralateral to the throwing arm ankle joint will present different ROM values compared to the ipsilateral ankle and that it would be related to vertical jump performance.

## 2. Materials and Methods

### 2.1. Participants

Fourteen young adult male handball players (19.3 ± 1.6 years, 1.86 ± 0.09 m, 78.7 ± 7.1 kg, 7.9 ± 3.8 years of playing experience), members of the U21 National Handball Team, participated in the study. Inclusion criteria were the systematic participation in the training program and the absence of a severe injury that deterred players from participating in their training and competition schedule for the past six months. Exclusion criteria were an inter-limb ankle joint ROM of >10°.

All participants provided a signed informed consent. The study was conducted following the guidelines of the Declaration of Helsinki and of the Institution’s Research Committee Ethics Code.

### 2.2. Experimental Procedure

To test the hypotheses of the study, the experimental procedure was conducted in two parts. In the first part, the active (ACT) and passive (PAS) ankle ROM was measured for both legs. The second part comprised the vertical jump tests. All tests were conducted on the same day that was after a day off from practice or a game.

#### 2.2.1. Range of Motion Measurement

ACT and PAS ankle ROM at knee extension angles (θ_KNEE_) of 0°, 40°, and 90° (0° = full extension) was measured using the video analysis method that is described in detail elsewhere [8]. The test was conducted in a random order concerning the ipsilateral (TAS) and contralateral (NTS) lower extremities. No warm-up was allowed prior to this measurement.

#### 2.2.2. Vertical Jump Tests

The vertical jump tests were executed in a random counterbalanced order. Warm-up consisted of 10 min cycling on an 817E Monark Exercise Cycle (Exercise AB, Vansbro, Sweden), 10 min of dynamic flexibility exercises, and a series of vertical jumps with progressively increasing intensity from sub-maximum to maximum.

The vertical jumps tests were performed on an AMTI OR6-5-1 force plate (AMTI, Newton, MA, USA). Ground reaction forces (GRF) were acquired at a nominal sampling frequency of 500 Hz. The following vertical jump tests were conducted:

1. Squat jump without an arm swing (SQJ-NAS): At the initial position, the feet were in full contact with the force-plate, and θ_KNEE_ was checked to be at 90° flexion [50]. The arms were kept on the hips during the impulse phase, the flight, and the landing.
2. Squat jump with an arm swing (SQJ-AS): The starting position of the lower extremities was the same as in the SQJ-NAS. The arms were hanging parallel to the side of the body and were swung upwards during the impulse phase.
3. Countermovement jump without an arm swing (CMJ-NAS): An upright starting position was adopted with the feet having full contact with the force-plate. The arms were positioned as described for the SQJ-AS. No limitations concerning the knee flexion during the countermovement were imposed.
4. Countermovement jump with an arm swing (CMJ-AS): The movement of the lower extremity was as in the CMJ-NAS. As for the arm swing, from a freely hanging position at the side of the body at the star, the upper extremities were swung backward and forward during the impulse phase.

Performance (h_JUMP_) was computed based on the body center of mass (BCM) vertical take-off velocity (Vy) that was extracted after the integration of the vertical GRF. The integration of Vy indicated the vertical BCM displacement from the initial starting position. Based on its lowest position (S_DOWN_), the downward and upward phases of the impulse were defined in the CMJs. The upward vertical BCM displacement (S_UP_) was measured from its lowest position until the take-off. The kinetic parameters examined were the maximum vertical GRF (Fz_MAX_), the maximum rate of force development during the upward phase (RFD_MAX_), and the peak power output (P_MAX_). In addition, temporal parameters were recorded, such as the total duration of the impulse phase (t_C_), the duration of the upward phase (t_UP_), as well as the time to achieve the Fz_MAX_ (t_Fz_) and the P_MAX_ (t_P_).

In all vertical jump tests, three maximal trials were performed. All attempts were executed barefooted. The instruction given was to “jump as high and as fast as possible”. A minimum of 60 s was allowed between trials to avoid fatigue and a 3 min interval separated each type of jumping test. For each vertical jump test, only the best attempt (criterion: highest h_JUMP_) was selected for further analysis.

The effectiveness of the arm swing (AS_EFF_) concerning h_JUMP_ was evaluated as shown in Equations (1) and (2) for the squat (SQJ) and countermovement (CMJ) jumps, respectively:

(1) $${\mathrm{AS}}_{\mathrm{EFF}\left(\mathrm{SQJ}\right)}=\frac{h_{\mathrm{JUMP}\left(\mathrm{SQJ}-\mathrm{AS}\right)}-h_{\mathrm{JUMP}\left(\mathrm{SQJ}-\mathrm{NAS}\right)}}{h_{\mathrm{JUMP}\left(\mathrm{SQJ}-\mathrm{NAS}\right)}}\times 100$$

(2) $${\mathrm{AS}}_{\mathrm{EFF}\left(\mathrm{CMJ}\right)}=\frac{h_{\mathrm{JUMP}\left(\mathrm{CMJ}-\mathrm{AS}\right)}-h_{\mathrm{JUMP}\left(\mathrm{CMJ}-\mathrm{NAS}\right)}}{h_{\mathrm{JUMP}\left(\mathrm{CMJ}-\mathrm{NAS}\right)}}\times 100$$

The respective effectiveness of the SSC imposed by the countermovement of the lower extremities (SSC_EFF_) in the arm (AS) and no arm swing (NAS) vertical jump tests was evaluated according to Equations (3) and (4):

(3) $${\mathrm{SSC}}_{\mathrm{EFF}\left(\mathrm{AS}\right)}=\frac{h_{\mathrm{JUMP}\left(\mathrm{CMJ}-\mathrm{AS}\right)}-h_{\mathrm{JUMP}\left(\mathrm{SQJ}-\mathrm{AS}\right)}}{h_{\mathrm{JUMP}\left(\mathrm{SQJ}-\mathrm{AS}\right)}}\times 100$$

(4) $${\mathrm{SSC}}_{\mathrm{EFF}\left(\mathrm{NAS}\right)}=\frac{h_{\mathrm{JUMP}\left(\mathrm{CMJ}-\mathrm{NAS}\right)}-h_{\mathrm{JUMP}\left(\mathrm{SQJ}-\mathrm{NAS}\right)}}{h_{\mathrm{JUMP}\left(\mathrm{SQJ}-\mathrm{NAS}\right)}}\times 100$$

### 2.3. Statistical Analysis

Data are presented as mean ± standard deviation. Normality of distribution and the equality of variance were assessed using the Shapiro–Wilk test (*p* > 0.05) and the Levene’s test (*p* > 0.05), respectively.

A 2 (TAS vs. NTS) × 2 (ACT vs. PAS) × 3 (0°, 40°, and 90° θ_KNEE_) MANOVA with repeated measures on the last factor after Bonferroni adjustments was run to test the effect of laterality, flexibility type, and knee angle on ankle ROM. Significant differences were followed up with simple contrasts and effect sizes were determined a posteriori using the partial eta-squared statistic (*η_p_*^2^). A 2 (countermovement; SQJ, CMJ) × 2 (arm swing: NAS, AS) repeated measures ANOVA with Bonferroni adjustment was used to examine the main effects of the stretch–shortening cycle, the arm swing, and their interaction on the kinetic and spatiotemporal parameters of the vertical jumps. Significant differences were followed up with pairwise comparisons. Effect sizes were checked using the partial eta-squared statistic (*η_p_*^2^). Small, medium, and large effect sizes were determined by the extracted values of above 0.01, 0.06, and 0.14, respectively [51]. The relationship of ACT and PAS ankle joint ROM at different θ_KNEE_ with h_JUMP_ and effectiveness indexes was checked using a two-tailed Pearson’s correlation analysis.

All statistical tests were conducted using the IBM SPSS Statistics v.27 software (International Business Machines Corp., Armonk, NY, USA). The level of significance was set at *α* = 0.05.

## 3. Results

Data from 12 players who met all criteria were included in the analysis. Their characteristics were the following: age: 19.1 ± 1.3 years; body height: 1.88 ± 0.09 m; body mass: 80.4 ± 6.2 kg.

### 3.1. Ankle Range of Motion

Table 1 depicts the results for the ankle ROM. ACT compared to PAS ROM was about 10.7° to 15.2° less across conditions. This resulted in a significant main effect of flexibility type assessment (*F*_1,157_ = 43.313, *p* < 0.001, *η_p_*^2^ = 0.220; large effect). Ankle ROM decreased as θ_KNEE_ extended from 90° flexion to full extension, indicating a significant knee joint angle effect (*F*_2,157_ = 10.928, *p* < 0.001, *η_p_*^2^ = 0.129; medium effect). Finally, the difference between TAS and NTS ranged from 3.5° to 9.3°. No significant inter-limb effect was observed (*F*_1,157_ = 3.218, *p* = 0.075, *η_p_*^2^ = 0.021; small effect).

### 3.2. Vertical Jump Tests

A significant countermovement (*F*_1,44_ = 12.045, *p* = 0.001, *η_p_*^2^ = 0.215) and arm swing (*F*_1,44_ = 7.237, *p* = 0.010, *η_p_*^2^ = 0.141) large effect was evident for h_JUMP_ (Table 2). A significant countermovement main effect was observed for Fz_MAX_ (*F*_1,44_ = 5.040, *p* = 0.030, *η_p_*^2^ = 0.103; medium effect size), S_DOWN_ (*F*_1,44_ = 299.045, *p* < 0.001, *η_p_*^2^ = 0.872; large effect size), S_UP_ (*F*_1,44_ = 6.723, *p* = 0.013, *η_p_*^2^ = 0.133; medium effect size), t_C_ (*F*_1,44_ = 24.355, *p* < 0.001, *η_p_*^2^ = 0.356; large effect size), t_UP_ (*F*_1,44_ = 55.839, *p* < 0.001, *η_p_*^2^ = 0.559; large effect size), and t_P_ (*F*_1,44_ = 26.621, *p* < 0.001, *η_p_*^2^ = 0.377; large effect size).

A significant arm swing main effect was revealed for P_MAX_ (*F*_1,44_ = 6.584, *p* = 0.014, *η_p_*^2^ = 0.130; medium effect size). Furthermore, a significant countermovement and arm swing interaction was observed for t_Fz_ (*F*_1,44_ = 14.939, *p* < 0.001, *η_p_*^2^ = 0.253; large effect size).

**Figure 1:**
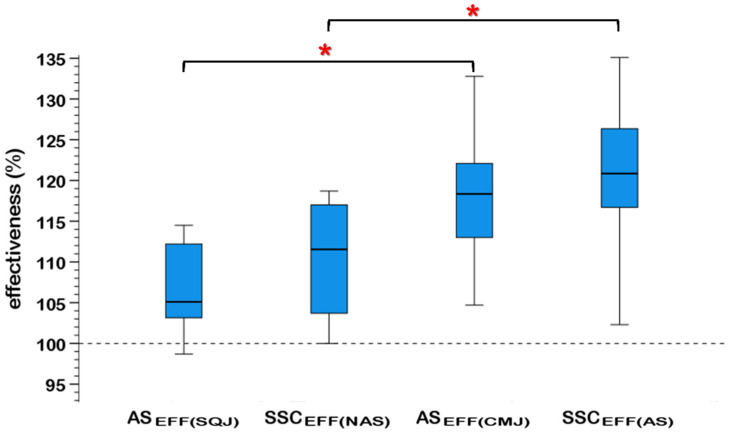
Effectiveness of the arm swing (AS_EFF_) and the countermovement (SSC_EFF_) on h_JUMP_ in the vertical squat (SQJ) and countermovement (CMJ) jumps (*n* = 12). The 100% mark represents the h_JUMP_ of the SQJ-NAS; *: *p* < 0.05.

With respect to the AS_EFF_ and SSC_EFF_, a significant main effect (*F*_1,44_ = 26.643, *p* < 0.001, *η_p_*^2^ = 0.377; large effect size) of the countermovement was observed (Figure 1). Neither arm swing main effect (*F*_1,44_ = 2.837, *p* = 0.100, *η_p_*^2^ = 0.060) nor a significant countermovement and arm swing interaction (*F*_1,44_ = 0.005, *p* = 0.943, *η_p_*^2^ = 0.000) were revealed.

The correlation analysis revealed that the only significant (*p* < 0.05) relationship between the TAS ankle joint ROM and vertical jump performance was for the ACT condition when the knee was fully extended. In specific, TAS ankle ACT ROM at θ_KNEE_ = 0° was moderately correlated with the h_JUMP_ measured for the SQJ-NAS (*r* = 0.65, *p* = 0.021), the SQJ-AS (*r* = 0.64, *p* = 0.024), and the CMJ-NAS (*r* = 0.69, *p* = 0.013).

## 4. Discussion

The present study tested the hypothesis that a lower ankle joint ROM would be observed in the contralateral compared to the ipsilateral to the throwing arm leg and that this difference would be related with the vertical jump performance of young adult handball players. The hypothesized inter-limb difference was not confirmed by the results of the study. However, there is evidence that the active ROM of the throwing arm side ankle joint was related to the majority of the vertical jump tests.

The results of the ROM measurements reveal no significant inter-limb differences except the active ROM when the knee was flexed at a 40° angle. The absence of an inter-limb difference confirms past findings concerning the flexibility [32,33,52,53] and isokinetic torque [54] of handball players. In general, no inter-limb ankle ROM differences have been reported in populations of various sports background [8,26,27,28,29,30,31]. This can be attributed to the fact that scientific evidence and recommendations aid in the design of training programs aiming to maintain symmetry in joint mobility [55,56,57] since it is widely acknowledged that decreased ROM is associated with sports injuries [58].

A significant main effect of the type of flexibility assessment was found [59]. This was clear for the ipsilateral to the throwing arm ankle, where the passive ROM of motion was significantly larger than the active ROM in all knee angles. This difference is in agreement with previous research findings concerning female handball players [60]. On average, a trend was noted regarding the lower ROM observed in the contralateral compared to the ipsilateral to the throwing arm ankle. This could be the result of the sport-specific demands of the shots in handball, where the former serves as the take-off leg. Take-off for the shot in handball is a strenuous task since a high loading on the musculoskeletal system occurs [45,61]. Loading, as expressed by its quantity, frequency, intensity, and magnitude of application, has an impact on the length and stiffness of the muscles acting on the working joint and thus alters its ROM based on the sport-specific demands imposed [32,62]. Bilateral asymmetries in vertical limb stiffness have been observed in the past, as stiff and compliant limbs can be identified in vertical jump testing [63]. These facts explain the findings of the present study. The absence of a significant difference between the active and passive ROM in the contralateral to the throwing arm ankle could be a result of the loading occurring during the shot take-off that leads to stiffer ankle extensor muscles. This results in decreased ankle dorsi flexion that leads to a reduced ROM, which is a common finding in handball players [60,64].

A significantly larger ankle ROM was recorded in the passive measurement condition when the knee joint was flexed from its full extension. This was also observed in a previous study on adolescent female volleyball players [8]. With the knee joint flexed at a 40° angle, the active ROM of the contralateral to the throwing arm ankle joint was approximately 70°. This is in agreement with previous findings [8,60,65] and confirms the notion that the flexibility of handball players is similar to those reported for other athletes [52]. The knee joint angle effect found is in line with previous findings [8,66]. Nevertheless, there is a bias in the literature, as passive ankle dorsi flexion ROM is suggested to be unrelated to knee joint angle [60,64,67,68]. Ankle dorsi flexion, which is the initial position of the ankle joint for a vertical squat jump, is affected by the extension rather than by the flexion of the knee joint as a result of the bi-articularity of the gastrocnemius muscle [69]. In addition, it is suggested that the active and passive lengthening of the gastrocnemius muscle is the major factor for controlling ankle dorsi flexion [70]. Thus, the altered angular position of the lower limb joints causes differentiation in the strength application capabilities of the shank muscles, as well as in the proximal-to-distal transfer of energy [71,72,73,74,75], which corresponds to the body segment sequencing for the execution of the handball throws [47,76,77].

A significant countermovement and arm swing effect were revealed in vertical jump tests, confirming past findings [5,6,8,78,79]. Nevertheless, a significant effect of the countermovement rather than the arm swing was found on the efficiency concerning jump height augmentation. This can be attributed to the fact that the arm swing contributes to higher torques about the ankle joint compared to the countermovement [78]. It is possible that the players with decreased ankle ROM were not able to take advantage of this. Since almost a quarter of the energy for the impulse is generated by the ankle joint [40,41], the observation that the arm swing was not effectively utilized in the participants examined in the present study could influence the previously mentioned finding. In general, restrictions in the ankle joint ROM were found to result in poor vertical jump performance [8,29,80,81]. The mechanisms responsible for this outcome are, firstly, the altered body configurations during the jump that results in a larger forward lean of the body and eventually in the inefficient utilization of the produced energy [29,43,82]; secondly, the limited upward vertical displacement leads to reduced work and power outputs that eventually limit the vertical body center of mass velocity [43] and, thirdly, there is a disruption of the energy flow through the gastrocnemius muscle [80]. In the present study, the aforementioned kinetic factors were found to be subjected to a main countermovement effect, justifying the suggested limited efficiency of arm swing due to the variance of the recorded ankle joint ROM.

The active ankle joint ROM of the ipsilateral to the throwing arm, when the knee was fully extended, was related to performance in the squat jump tests, as well as in the countermovement jump without an arm swing. Past research suggests that the optimum knee angle for the highest strength application capability is about 40° flexion [72,83]. However, it has been found that when the ankle is dorsiflexed and the knee joint is concurrently fully extended, the gastrocnemius muscle is able to generate the highest plantar flexion moment [84]. In addition, it is evident that when aiming to perform a throw with a jump, handball players tend to rely on the rapid execution of the propulsive phase of the support in a horizontal direction [45,85]. Furthermore, the knee angle of the lead leg is almost at near extension at the instant of take-off [47]. This leads to a lower limb configuration with an almost extended knee and a plantarflexed ankle to absorb the energy at the touchdown [85]. As this is repeatedly executed, it is expected to result in the ability of handball players to manage torques of high magnitude around the ankle joint when the knee is extended as a sport-specific adaptation [32,86]. Nevertheless, the above-mentioned lower limb configuration is a contributing factor to Anterior Cruciate Ligament injury [87,88]. Thus, it is recommended to include jumping and landing exercises as part of preventive training programs for handball players [89].

This study is not without limitations. The relationship between vertical jump performance and ankle joint range of motion was examined using bilateral, instead of unilateral, jumping tests. The option to use bilateral jumping tests was promoted due to the wider consideration and application of the standardized vertical jumping tests to check the hypothesis of the study [90], despite the considerations raised for their usage [91]. The same rationale was adopted to exclude the usage of the jump shot per se as the criterion for evaluating the jumping ability of the participants. A second limitation is the absence of controlling the depth of the countermovement since self-selected downward velocity and joint flexion cause inter-individual differences in the biomechanics of the CMJ [92]. In addition, no joint angular kinematics were acquired to establish the ankle joint range of motion during the vertical jump tests and its contribution in terms of joint work and power. Finally, the inter-limb differences concerning morphological structure (i.e., bone structure and deformities of the ankle joint and the feet, muscle mass, etc., which are muscle architecture features) were not evaluated. Thus, future research should establish research protocols examining the relationship of flexibility and body structure measures with sport-specific jumping tests in handball players. Added to that, the relationship of the ankle joint range of motion in clinical flexibility tests with the respective during the execution of handball technique elements (i.e., jump shot) should be examined. Furthermore, the possible inter-limb difference of joint range of motion and its effect on sport-specific movements in reference to the player position could also be of interest to investigate.

## 5. Conclusions

The ankle joint range of motion was found not to be different between the throwing and non-throwing side of handball players. Nevertheless, the passive ankle range of motion was larger than the active range of motion. In addition, the ankle joint range of motion decreased as the knee joint increased. The reported values of ankle range of motion related to knee joint flexion and handedness can be used by practitioners, coaches, athletic trainers, and physiotherapists when designing training and rehabilitation programs.

In addition, the kinetic parameters of vertical jumping were differentiated when a countermovement, an arm swing, or both were utilized, with the former being the most significant factor for the increment in jump height among the mechanisms tested for the augmentation of vertical jump performance. An indication that ankle joint range of motion when the knee was extended is related to jumping performance in the examined handball players. This should be taken under consideration by coaches and practitioners when including vertical jumps in training. Differences in the segmental positioning of the lower limbs, especially the modifications concerning the relative position of the ankle and knee joints at take-off and landing from a jump shot can result in alterations in tissue loading. Thus, flexibility and proprioception tests should be implemented regularly.

In conclusion, a goal of training programs in handball training should be the improvement of the ankle range of motion. This could be beneficial for augmenting the effectiveness of the use of the arm swing in vertical jumping and, thus, might improve the proximal to distal energy transfer that is evident in the jump throw.

## Figures and Tables

**Table 1:** Results for the ankle joint range of motion measurements (*n* = 12).

	θ_KNEE_ = 0°	θ_KNEE_ = 40°	θ_KNEE_ = 90°
ROM Measurement	Mean ± SD	Mean ± SD	Mean ± SD
TAS			
ACT (deg)	52.7 ± 11.7	56.2 ± 9.6	63.4 ± 18.0
PAS (deg)	69.0 ± 10.9 *	74.2 ± 13.4 *	79.3 ± 13.2 *
NTS			
ACT (deg)	59.4 ± 6.3	65.4 ± 10.0 ^#^	70.4 ± 10.5
PAS (deg)	65.3 ± 9.7	78.0 ± 12.6 *^,a^	76.8 ± 13.5 ^a^

^a^: significantly different compared to θ_KNEE_ = 0° (*p* < 0.05); *: significantly different compared to ACT (*p* < 0.05); ^#^: significantly different compared to TAS (*p* < 0.05); ROM: range of motion; θ_KNEE_: knee joint angle; TAS: ipsilateral to throwing arm; NTS: contralateral to throwing arm; ACT: active flexibility range of motion test; PAS: passive flexibility range of motion test.

**Table 2:** Results (*n* = 12) of the kinetic and spatiotemporal parameters for the vertical squat (SQJ) and countermovement (CMJ) jump with (AS) and without the use of an arm swing (NAS).

Parameter	Arm Swing	SQJ	CMJ	Countermovement Effect		Arm Swing Effect		Interaction	
		Mean ± SD	Mean ± SD	*p*	*η_p_* ^2^	*p*	*η_p_* ^2^	*p*	*η_p_* ^2^
h_JUMP_	NAS	27.3 ± 3.9	30.0 ± 4.5	0.001	0.215	0.010	0.141	0.183	0.040
(cm)	AS	29.0 ± 3.6 ^a^	35.3 ± 5.8 ^a,c^						
Fz_MAX_	NAS	2.3 ± 0.2	2.5 ± 0.3	0.030	0.103	0.449	0.013	0.958	0.000
(N/kg)	AS	2.3 ± 0.3	2.5 ± 0.2						
RFD_MAX_	NAS	8.2 ± 2.4	11.6 ± 4.1 ^c^	0.073	0.071	0.818	0.001	0.078	0.069
(kN/s)	AS	10.1 ± 2.8	10.1 ± 3.3						
P_MAX_	NAS	27.1 ± 5.2	26.7 ± 5.7	0.107	0.058	0.014	0.130	0.064	0.076
(W/kg)	AS	28.3 ± 6.3	34.4 ± 6.8 ^a,c^						
S_DOWN_	NAS	0.0 ± 0.0	−19.6 ± 4.2 ^a^	<0.001	0.872	0.969	0.000	0.311	0.023
(% body height)	AS	0.0 ± 0.0	−18.6 ± 4.1 ^a^						
S_UP_	NAS	28.2 ± 2.1	31.0 ± 4.1	0.013	0.133	0.229	0.033	0.991	0.000
(% body height)	AS	29.5 ± 4.0	32.3 ± 4.5						
t_C_	NAS	510 ± 114	626 ± 76 ^c^	<0.001	0.356	0.804	0.001	0.586	0.007
(ms)	AS	489 ± 103	577 ± 77 ^c^						
t_UP_	NAS	100 ± 0	52 ± 3 ^c^	<0.001	0.559	0.735	0.003	0.881	0.001
(%t_C_)	AS	100 ± 0	53 ± 3 ^c^						
t_Fz_	NAS	67 ± 11	50 ± 7 ^c^	0.477	0.012	0.075	0.070	<0.001	0.253
(%t_C_)	AS	60 ± 15	71 ± 16 ^a,c^						
t_P_	NAS	78 ± 5	82 ± 2 ^c^	<0.001	0.377	0.614	0.006	0.440	0.014
(%t_C_)	AS	76 ± 4	83 ± 3 ^c^						

^a^: significant arm swing effect (*p* < 0.05); ^c^: significant countermovement effect (*p* < 0.05); h_JUMP_: jump height; Fz_MAX_: maximum vertical ground reaction force; RFD_MAX_: maximum rate of force development in the upward phase; P_MAX_: peak power output; S_DOWN_: vertical body center of mass displacement during the downward phase; S_UP_: vertical body center of mass displacement during the upward phase; t_C_: total duration of the impulse; t_UP_: duration of the upward phase; t_Fz_: time to achieve Fz_MAX_; t_P_: time to achieve P_MAX_.

## Data Availability

The data that were used in the present study can be provided by the corresponding author upon reasonable request.
